# Stakeholder perspectives on social screening in US healthcare settings

**DOI:** 10.1186/s12913-023-09214-z

**Published:** 2023-03-13

**Authors:** Benjamín Aceves, Emilia De Marchis, Vishalli Loomba, Erika M. Brown, Laura M. Gottlieb

**Affiliations:** 1 School of Public Health, San Diego State University, 5500 Campanile Drive, 92182 San Diego, CA USA; 2 Department of Family & Community Medicine, University of California, San Francisco, 995 Potrero Ave, 94110 San Francisco, CA USA; 3 Social Interventions Research and Evaluation Network, University of California, San Francisco, 3333 California Street, Suite 465, 94118 San Francisco, CA USA

**Keywords:** Social care, Screening, Health services research, Qualitative research, Health equity

## Abstract

**Background:**

Evidence on the health impacts of social conditions has led US healthcare systems to consider identifying and addressing social adversity—e.g. food, housing, and transportation insecurity—in care delivery settings. Social screening is one strategy being used to gather patient information about social circumstances at the point of care. While several recent studies describe the rapid proliferation of social screening activities, little work has explored either why or how to implement social screening in clinical settings. Our study objectives were to assess diverse healthcare stakeholder perspectives on both the rationale for social screening and evidence needed to inform practice and policy-relevant implementation decisions.

**Methods:**

We convened five focus groups with US experts representing different stakeholder groups: patient advocates, community-based organizations, healthcare professionals, payers, and policymakers. In total, 39 experts participated in approximately 90-minute long focus groups conducted between January-March 2021. A inductive thematic analysis approach was used to analyze discussions.

**Results:**

Three themes emerged from focus groups, each reflecting the tension between the national enthusiasm for screening and existing evidence on the effectiveness and implementation of screening in clinical settings: (1) ambiguity about the rationale for social screening; (2) concerns about the relavence of screening tools and approaches, particularly for historically marginalized populations; (3) lack of clarity around the resources needed for implementation and scaling.

**Conclusion:**

While participants across groups described potential benefits of social screening, they also highlighted knowledge gaps that interfered with realizing these benefits. Efforts to minimize and ideally resolve these knowledge gaps will advance future social screening practice and policy.

**Supplementary Information:**

The online version contains supplementary material available at 10.1186/s12913-023-09214-z.

## Introduction

**Fig. 1:**
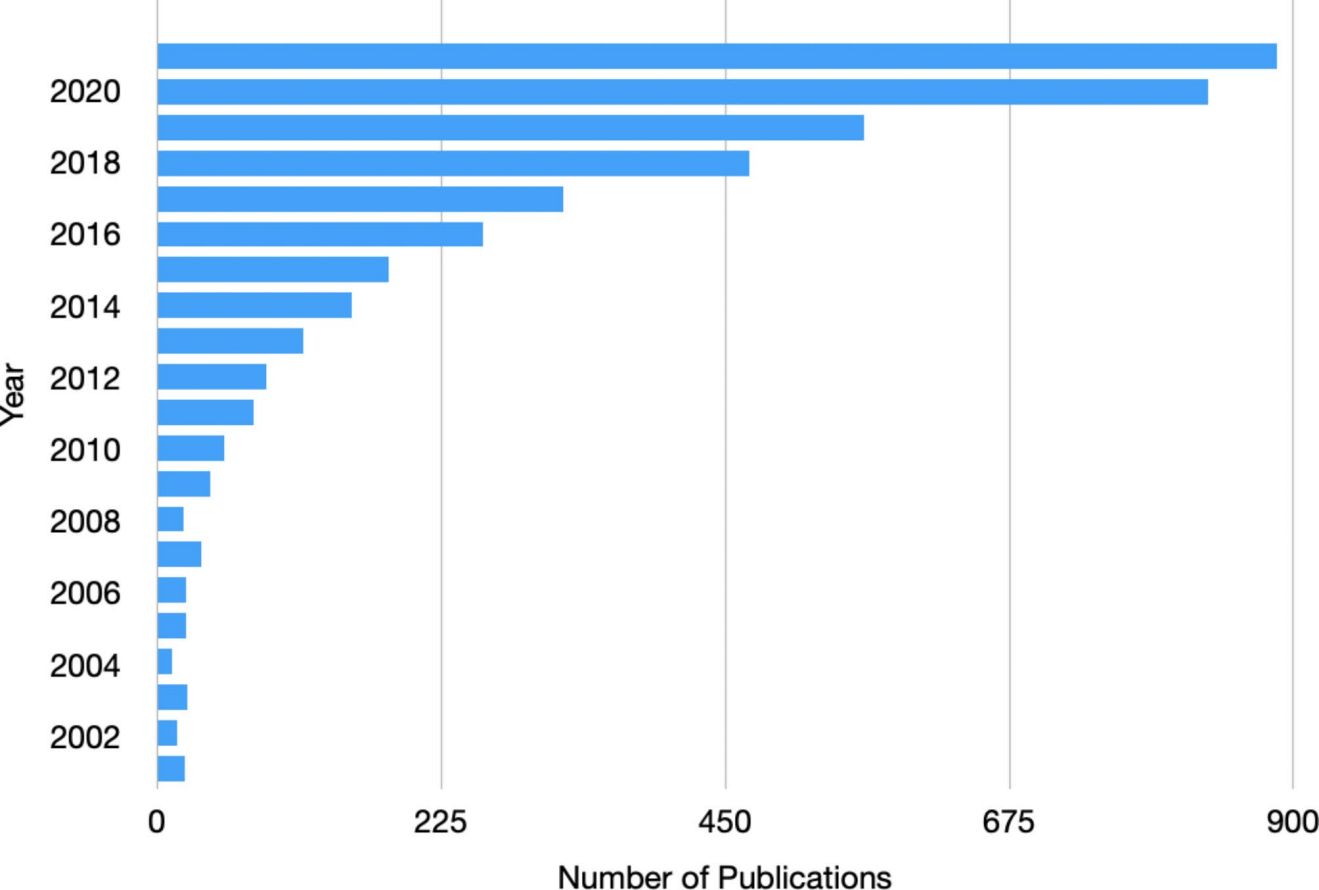
Number of social screening-related publications by year, 2001–2021. Source: PubMed search on June 8, 2021. The search used the following terms: (“Social Determinants of Health“[Mesh] OR “social determinants” OR “social determinant” OR “social needs” OR “social need” OR “social risk” OR “social risks”) AND (“Mass Screening“[Major] OR screening OR screen OR “needs assessment " OR “need assessment”)

Substantial and compelling evidence documenting the health impacts of patients’ social conditions—including food, housing, and transportation security—has led many US healthcare systems to more routinely consider patients’ social conditions in the context of health care delivery. Social risk and asset screening (“social screening”) at the point of care has emerged as a cornerstone of burgeoning initiatives that aim to use information about patients’ social conditions to inform medical treatment and management decisions, referrals to social services, and more broadly, health care sector investments in community resources [1]. As a result, multiple federal agencies and medical professional organizations have developed or otherwise endorsed social screening tools [1, 2]. The majority of these tools include questions related to food, housing, and transportation security [2, 3]. As enthusiasm for screening initiatives grows, a new crop of research studies on social screening has emerged, often describing the reach of social screening in select settings or populations. (See Fig. 1). Despite the apparent increase in social screening activities, no clear consensus has emerged on why, when, or how to design and implement screening programs in US clinical settings [4–6].

We convened five distinct groups of healthcare stakeholders to better understand stakeholder perspectives on the rationale for screening and evidence that would support implementation decision-making. Each group represented a different stakeholder type involved in social screening and related interventions. In this paper we describe findings from our discussions with these stakeholders.

## Methods

We recruited a convenience sample of US experts in patient advocacy, community-based social services delivery, healthcare practice, healthcare payors, and policy using a snowball sampling strategy that drew from the research team’s existing national network on health and social care. These experts represented stakeholders from across the US, who have experience in addressing health disparities among marginalized populations. We asked the initial sample of contacts to recommend colleagues from diverse areas of the country whom they considered experts in the same content areas. From the solicited recommendations and the research team’s known contacts, we emailed 58 potential expert stakeholders with information about study aims, specific focus group objectives and activities, and the estimated time commitment for study participation. In cases where participants identified with more than one topic, each participant was asked to choose their primary field of expertise (e.g. payment vs. policy) and was assigned to one of five stakeholder groups based on their stated preference: patient advocates, community-based organization (CBO) leaders, healthcare professionals, healthcare payers, and healthcare policy-makers.

The five 90-minute video focus group calls took place between January and March 2021, each via a university-sponsored Zoom meeting platform. Participants provided verbal consent to participate at the onset of each session; each was offered a $50 gift card in recognition of their contribution. Each session was moderated by a study team member with experience conducting focus groups (L.M.G.), who guided conversations using a semi-structured focus group guide. (Full text of semi-structured focus group guide is available in Appendix 1.) The interview guide was developed by the research team based on their collective prior research on social screening in healthcare settings. The interview guide explored participants’ perspectives on the rationale for screening and evidence that would support implementation decision-making, including strategies to achieve equity in screening practices and patient experience of screening—a copy was provided to participant prior to focus group sessions. Sessions were audio recorded and audio files were stored in a secure database. Recordings were transcribed using the online service Rev.com. All study activities were approved by the Institutional Review Board at the University of California, San Francisco.

### Analytical approach

Using Dedoose software to facilitate analysis, we applied a inductive thematic approach to interpret stakeholders’ perspectives on social screening [7]. All study team members independently reviewed the same randomly selected transcript and developed a preliminary analytic codebook structured to explore themes related to the rationale for screening and evidence needed/missing to inform practice and policy. Two research team members trained in qualitative research methods (B.A., E.D.) then used the initial codebook to independently and more deeply analyze and code a second randomly selected transcript. These two team members then discussed emergent themes, discrepancies, revised the codes, and established codebook agreement. Each of the remaining transcriptions was subsequently analyzed and reviewed independently by three team members (B.A., E.D., V.L.), who met regularly to resolve any coding discrepancies. Themes were identified from the codes and discussed. The emerging themes were presented to the other research team members (E.B., L.M.G.) for further development.

## Results

Thirty-nine of 58 (67%) of the experts we approached agreed to participate in the study, including eight patient advocates, five CBO leaders, nine health care professionals, eight payers, and nine policymakers. Three key themes emerged across the five stakeholder group conversations and are described below.

### Ambiguity about the rationale for social screening

Participants shared multiple pathways through which social screening in healthcare settings might improve health and advance health equity. These arguments included the potential for screening itself to improve the experiences and outcomes of patients experiencing socioeconomic barriers by enhancing relationships between these patients and their care teams. They also described how information derived from screening could inform interventions to personalize medical care and facilitate connections to needed social services, both of which were anticipated to improve health and reduce avoidable health care utilization. Screening was portrayed as a necessary first step towards using social data to achieve these outcomes:

I feel at the most basic level, health care organizations can’t address the needs if they don’t know they exist. You can assume inequities, but you can’t really pinpoint them or know who they are…unless you ask in some way, shape, or form. – Health care professional.

Other benefits of screening were described at the systems level, including the potential for data collection to contribute to better data-sharing between health, public health, and CBOs, which would lead to a more comprehensive understanding of health disparities and, ideally, more funding for health and social services in historically disadvantaged communities. A CBO participant described the potential community-level benefits of social data collection:

I think it informs the communities, the broader communities, about where there may be unmet need, right? That the community needs to be aware of, right? So I do think that it’s important to have some sense of the population, right? So that communities can figure out how to address those needs.

While enumerating the theoretical benefits of screening, each of the five discussions also elevated concerns about whether these potential benefits could be realized through healthcare-based initiatives. The most common concern was whether screening and efforts to link patients with social services would realistically contribute to a measurable increase in the receipt of social services and subsequent reduction in patients’ experiences of social adversity. Participants were concerned that screening might instead contribute to harm—such as increased patient distrust in the healthcare system and/or perceptions of discrimination—if the healthcare team did not provide meaningful assistance around identified social barriers. A patient advocate participant noted: “We don’t really have a system built in that actually guarantees the information that folks share [will be addressed].” This concern about helping to resolve patients’ needs led focus group participants to wrestle with whether the rationale for social screening actually rested on connecting patients with social services.

Participants went on to deeply question the motivations and assumptions behind the rationales for screening. Participants in the CBO group lacked confidence that healthcare activities around social care would drive systems change. As one CBO participant noted, “…should the healthcare system be the ones that collect that data? Because the reality for me then is that, who does that data then benefit?”

A patient advocate participant challenged health systems to more clearly articulate the goals of each screening initiative. Uncertainty about the primary rationale for screening made it more challenging for participants in each group to feel like they could make informed decisions about implementation. For instance, if the rationale for a screening initiative were primarily to connect patients with services, participants noted that the design of the screening program should prioritize the availability of effective social services. If the rationale for a screening program were instead to strengthen patient-provider relationships, then the design of the screening program should prioritize training for teams conducting screening to improve the screening experience for patients. Without a clear primary rationale, critical program design elements were also ambiguous.

### Concerns about the relevance of screening tools and approaches, particularly for historically marginalized populations

Across the five focus groups, participants questioned the revalence of different assessment tools and approaches. This was reflected in group discussions about the utility of standardized screening tools versus the benefits of customization that might make tools more acceptable to specific populations and contexts.

Payers and policymakers were more likely to see advantages to using standardized tools to ensure social data were both reliable and aggregatable, though they also recognized the potential advantages of customization. One policymaker participant shared:

You’re only as good as the information you get. You can standardize all you want, but if people are not understanding or it doesn’t feel appropriate or relevant, it’s sort of worthless.

Arguments for standardization included that common measures could facilitate the data aggregation needed to justify increased funding to serve marginzalized populations. Several participants more deeply appreciated the potential benefits of tailored approaches to screening based on race, ethnicity and language (REL) and social determinants of health (SDH), including relationship-centered or conversational approaches to gather information about patients’ social context.

We need to tailor messages really based off of REL in order to have an impact and then SDH, certainly that will be pulled in as well. I do think it helps. You need to understand your population, and how to deliver those messages, because if you don’t, and you’re asking a lot of personal questions related to SDH, that’s where you get some of that mistrust from your population. - Payer

Group conversations related to approaches were not limited to different modes (e.g. screening conducted via questionnaires versus in person by a member of the healthcare team) and models for screening (e.g. use of standardized tools versus more personalized approaches) but also extended to the content of screening. For instance, in patient advocate, policymaker, and healthcare professional focus groups, participants raised questions about whether strengths-based assessment tools exist and might better serve historically marginalized populations. Participants also noted that the existing screening tools lack content relevant to making meaningful changes to care: “Some of the resources that people may need are not in some of those existing tools. They may not include things for spiritual services, traditional services, culturally-based services, language.” –Policymaker.

In all discussions, participants expressed concerns that screening is likely to be perceived differently by people who have experienced racism and discrimination—and that being asked about or disclosing social risks might exacerbate patients’ feelings of stigma or marginalization. Participants lacked clarity around whether these potential negative consequences would be outweighed by positive consequences, including that the collected data could be used in ways that would reduce the effects of systemic racism and discrimination. With the goal of reducing negative experiences of screening for historically marginalized populations, some participants expressed interest in evidence on the psychometric and pragmatic properties of screening tools for specific sub-populations [8]. More commonly, however, participants focused less on psychometric and pragmatic properties and instead on the need for trauma-informed approaches to social screening, including data collection mechanisms that build, rather than erode, trust. As an example, participants noted that healthcare sector-based screening might mean duplicative data collection, in this case meaning multiple agencies would collect the same patient data, which at best would require more time from patients and service agencies and at worst could retraumatize people already experiencing social marginalization.

One patient advocate emphasized that best practice models on trauma-informed social screening already exist but need to be applied more routinely to screening practices:

[There] are groups that they’re under resourced and are small, but they’ve managed to be really successful and building trust and building relationships and getting the information they need to help their patient’s holistic needs.

### Lack of clarity around the resources needed for implementation and scaling

A third major theme that emerged in the stakeholder discussions was related to uncertainty around the infrastructure and resources needed for implementing and scaling screening initiatives. As an example, participants indicated that more information was needed about how many and which staff would be responsible for administering screening and responding to results. Anticipated workforce concerns extended to doubts that CBO partners could manage the influx of referrals from healthcare systems.

Questions about capacity also included other resource needs for screening along with the workforce. Participants’ comments underscored the intersection between screening and interventions.

Is there infrastructure within the healthcare system? Is there a technology platform that houses resources? Are the resources there? Are the workers train[ed] and connecting patients to those resources? Are the people asking those questions? Are they trained in culturally and linguistically appropriate services? What format are these questions being asked? Is it language accessible? - Patient advocate.

Participants noted that as screening workflows emerged, new team trainings would also be needed to implement and sustain them.

Lack of clarity about internal best practices mirrored participants’ concerns about external factors that also would influence program implementation and scaling. For example, participants noted that funding social service initiatives through grants—whether to healthcare or social service organizations—made it challenging to sustain services. Payer and healthcare professional groups, however, noted that more sustainable payment models were not yet in place because of the lack of return on investment (ROI) data. One payer participant emphasized: “It’s scalability and sustainability, and you get to that through health outcomes and ROI. That’s really what it comes down to.” Another payer participant added:

Many plans are paying for this work out of their reserves or with private funding, or through partnerships. What happens? Are these efforts sustainable? Particularly when they haven’t been studied in terms of their outcomes very much. It could potentially be a perfect storm. Decreased state funding, no federal guidance that assures Medicaid dollars for this, and then very few outcomes evaluations that show that these [programs] are effective.

## Discussion

As the US healthcare sector weighs its roles and responsibilities around reducing social adversity to improve health and health equity, delivery organizations are more actively implementing social screening [9], and payers and accreditation agencies are increasingly considering new screening quality measures to support implementation [10]. But neither why nor how to implement screening has been clearly articulated. This is the first study of which we are aware to explore the perspectives of five unique stakeholder groups—including patient advocates, CBOs, health care professionals, payers, and policymakers—on social screening in US healthcare settings. With only minor differences in emphasis, 39 participants across these groups highlighted common areas of uncertainty that will need to be explored before scaling screening.

Our participant discussions suggest that some of the uncertainty could be resolved with better evidence on the impact of screening (and related interventions) and then on the implementation of effective programs. More robust ROI data are needed, including data on patient experience, health, and health care utilization outcomes. Even as research on these impacts has increased over the last decade—the heterogeneity of evidence has made it difficult to interpret and synthesize results [11, 12]. Stakeholders particularly underscored the importance of more rigorous research on the impacts of social screening in diverse—historically marginzalized populations. This was reflected in their concerns about tool content and screening approaches in different sub-populations. If screening is at least in part about redressing inequities, screening practices must be designed in ways that minimize and ideally avoid unintended negative consequences, such as retraumatizing patients and/or exacerbating distrust in patients who have experienced discrimination and other forms of racism in healthcare settings. All focus groups emphasized the need to strengthen evidence connecting patient-centered and trauma-informed care—which have been shown to improve provider-patient relationships and lower patient distress when applied to other content areas [13–15]—with social screening, specifically. In this area, the healthcare system might apply learnings from CBO-based work on screening and intervening on social adversity [16].

Focus group participants also shared their uncertainty about the healthcare sector’s overall capacity for implementing new social care practices. Their considerations primarily revolved around workflows and workforce, technology, and funding needed to support screening and any subsequent interventions. While there are a growing number of consensus recommendations for implementing healthcare-based screening initiatives [17–21], these reports often rely on expert and stakeholder perspectives more than a rigorously developed evidence base [5, 22, 23]. The implementation evidence is gradually improving, however. Recent studies suggest provider education and training, for instance, can influence screening adoption [24–28]. Hybrid effectiveness-implementation studies offer one strategy for simultaneously strengthening the evidence base on implementation and examining the impacts of social screening programs [29–32].

In parallel, however, our findings should prompt a more in-depth national conversation about the goals behind the more deliberate integration of social and medical care services. Defined goals should drive program design and delivery. For instance, if the primary goal of screening is connecting patients with social services, screening should be accompanied by targeted, intentional investments in social services interventions. Accountability for those types of process outcomes would be appropriate and should be measured, e.g. healthcare systems would need to more routinely assess the availability of services and establish social services partnerships relevant to population needs.[33] Goals also should be used to shape payment demonstrations and related federal and state policy decisions [9]. In another example, if screening is primarily expected to inform health systems’ population-level investments, accountability metrics should be designed to assess changes in those investments [34,35].

Patients/consumers, practitioners, payers, and ethicists can together articulate the primary goals of screening programs and define the related suite of accountability metrics. This will help to ensure that care integration activities are not only designed to meet their goals but also feasible to implement and acceptable to key stakeholders. This kind of stakeholder-informed consensus-making process might be led by the US Office of the Assistant Secretary for Planning and Evaluation, an agency that already has been actively involved in exploring the health care sector’s potential roles in providing social care [11, 36, 37].

Findings from this qualitative research should be considered in light of several limitations. Stakeholders were selected using a snowball sampling design that contributed to participant selection bias. In addition, while our team did attempt to gather participants with a wide range of perspectives, racial, gender, and other important demographich data were not collected. The five different stakeholder groups nonetheless reflected a wide range of expertise and perspectives. Since the goal of this formative research was to better understand stakeholder perpsectives, this limitation provides no reason to discount the validity of the participants’ suggestions. We also recruited several participants who represented national organizations, some of whom had not been or were not at the time involved in screening initiatives. These participants may have been less familiar with common facilitators, barriers, and other on-the-ground challenges to screening, though they brought other nationally-relevant content to the discussions. Finally, focus group formats can influence participants’ willingness to disclose potentially controversial or negative perspectives. To reduce the likelihood of social desirability bias, we designed the discussion guide explicitly to solicit input on both facilitators and barriers to screening, which led to rich conversations about barriers to implementation.

## Conclusion and future directions

While consensus emerged about the theoretical benefits of screening, participants underscored multiple barriers to realizing those benefits, the most foundational being questions about the primary rationale for screening initiatives. Coupling conversations about the ethics of and evidence behind social screening may better inform the future design, implementation, and scaling of screening programs within the US.

## Electronic supplementary material

Below is the link to the electronic supplementary material.

Supplementary Material 1

## Data Availability

The datasets used during for this study are not publicly available nor available from the corresponding author because of the qualitative nature of the focus group data and the inability to de-identify the data. For any request please contact the corresponding author, BA.

## Abbreviations

CBO: Community-based organizations
REL: Race language, and ethnicity
ROI: Return on investment
SDOH: Social determinants of health
