# Supplementary material for: Stakeholder perspectives on social screening in US healthcare settings
Source: BMC Health Serv Res. 2023 Mar 13;23:246. doi: 10.1186/s12913-023-09214-z (PMC10012605; doi:10.1186/s12913-023-09214-z)
Supplement: Supplementary file 1 — Supplementary Material 1 [file 12913_2023_9214_MOESM1_ESM.pdf]

Appendix 1: Discussion Guide for SCREEN Study Advisory Panels

Introductions: Share names/affiliations
Also see Moderator Guide for SCREEN Study Advisory Panel Discussions for detailed introduction and setting of ground rules

Overview: The health care sector is increasingly adopting – or at least strongly considering – initiatives to facilitate systematic screening for social risk factors in the context of clinical encounters. With support from the Robert Wood Johnson Foundation, our research team is planning a comprehensive review of the literature on social risk screening efforts to better understand the existing evidence on both the effects of screening and facilitators and barriers to implementation. We are holding focus groups with a range of stakeholders to first ensure we ask the right questions in our review and then later in the year, we will return to you all to ask for feedback on review findings. Today, we really just want to hear your opinions about what health care systems need to know before making decisions about launching or scaling social risk screening programs.

When we mention to “social risk screening in clinical encounters” we are referring to ways that health care systems are using standardized screening tools to assess patients’ social risks factors. These tools may be adopted/adapted from another organization (e.g. the National Association of Community Health Centers PRAPARE tool) or a unique survey developed by that health system. We are focused on tools that include questions about topics related to social and economic domains like: housing stability and quality, food insecurity, utilities security, transportation, personal safety, educational attainment, literacy, financial strain, employment, immigrant/documentation status, internet/technology access, incarceration history.

Q1. What kinds of information do you think would be most valuable in helping to decide whether to launch or expand social risk screening programs?

- Can you think of any examples where you think existing research/evidence has already influenced the uptake or effectiveness of social risk screening programs in health care?
- Where do you think (more) information about what works is needed to advance these efforts?
- How would you prioritize these different areas where you think we need more information?

Q2. We want to zero in a little on existing evidence exploring ways social risk screening might affect health equity. We are using the Robert Wood Johnson Foundation’s definition of health equity: “that everyone has a fair and just opportunity to be as healthy as possible.”

- What equity-related outcomes are most important to you and/or your organization?
    - Are you aware of work that has explored the potential unintended consequences of social risk screening?
- Are you familiar with work (whether from your own org or other orgs) on the equity impacts of social risk screening? If so, how were equity impacts assessed in that work?

Q3. Where do you and your colleagues search or where would you consider searching for information or evidence about health care-based social risk screening initiatives?

- If academic research, which sources do you look to?

- If non-academic sources, where do you/would you look to inform decisions to screen/expand screening patients in health care settings?

Q4. Overall, what is your sense of what the current gray and academic literature conclude about social risk screening programs?

- Does anything feel like it is missing from the literature?
    - For instance, do we feel like you need to know more about the impacts of social risk screening initiatives? What more do you need to know?
    - For instance, do you feel like you need more information about the implementation of social risk screening initiatives? What more do you need to know?
- If you had to pick just one critical focus for future research on health care-based social risk screening, what would it be and why?
    - If we had asked you this same question prior to the COVID pandemic, do you think you would have had a different answer? Why?

Q5: As we head off to search the literature in this area, is there anything else we should be looking for that would be helpful for you, patients, providers, and health systems you work with?
